# Silencing of hsa_circ_0101145 reverses the epithelial-mesenchymal transition in hepatocellular carcinoma via regulation of the miR-548c-3p/LAMC2 axis

**DOI:** 10.18632/aging.103324

**Published:** 2020-06-18

**Authors:** Jinglan Jin, Huan Liu, Meishan Jin, Wanyu Li, Hongqin Xu, Feng Wei

**Affiliations:** 1 Department of Hepatology, The First Hospital of Jilin University, Changchun 130021, China; 2 Department of Hepatobiliary and Pancreas Surgery, The First Hospital of Jilin University, Changchun 130021, China; 3 Department of Pathology, The First Hospital of Jilin University, Changchun 130021, China

**Keywords:** hsa_circ_0101145, epithelial-mesenchymal transition, hepatocellular, miR-548c-3p, LAMC2

## Abstract

Hepatocellular carcinoma (HCC) is a primary cause of cancer-related deaths globally. While there have been advancements in HCC treatment and diagnosis, incidence and mortality rates continue to rise. One study found that circular RNAs functioned as competing endogenous RNAs, and constructed a gene-based nomogram to estimate overall survival of HCC patients. Previous studies using high-throughput sequencing suggested that hsa_circ_0101145 is abnormally expressed in HCC, but the underlying mechanism is unknown. We performed RT-qPCR to determine hsa_circ_0101145 and miR-548c-3p expression in HCC tissues. We used fluorescence *in situ* hybridization (FISH) to detect hsa_circ_0101145 expression and hsa_circ_0101145 subcellular localization in HCC tissues. hsa_circ_0101145 expression in HCC cells was selectively regulated. We determined LAMC2 and EMT mRNA and protein levels by RT-qPCR and western blotting analysis, respectively. We employed flow cytometry, and CCK8, Transwell, and wound healing assays to monitor the cell cycle, cell proliferation, invasion, and migration, respectively. We employed dual-luciferase reporter and RNA pulldown assays to verify the relationship among hsa_circ_0101145, miR-548c-3p, and LAMC2. We examined the effects of hsa_circ_0101145 on HCC cell metastasis and proliferation *in vivo* using a subcutaneous xenograft model as well as intravenous tail injection of nude mice. The data demonstrated that hsa_circ_0101145 was significantly upregulated in both HCC tissues and cell lines. High hsa_circ_0101145 expression was correlated with aggressive HCC phenotypes. Downregulation of hsa_circ_0101145 suppressed HCC proliferation as well as metastasis by targeting the miR-548c-3p/LAMC2 axis, which was examined using luciferase reporter and RNA pulldown assays. Silencing of hsa_circ_0101145 suppressed the epithelial-mesenchymal transition in HCC. Downregulation of miR-548c-3p or overexpression of LAMC2 restored migration and proliferation abilities of HCC cells following hsa_circ_0101145 silencing. LAMC2 overexpression reversed miR-548c-3p-induced cell migration and growth inhibition *in vitro*. In summary, the findings illustrated that hsa_circ_0101145 silencing suppressed HCC progression by functioning as an miR-548c-3p sponge to enhance LAMC2 expression. Therefore, hsa_circ_0101145 could be an HCC treatment target.

## INTRODUCTION

Hepatocellular carcinoma (HCC) is the fifth most common malignancy and the second leading cause of cancer-related mortality worldwide [1]. Currently, the main treatments for liver cancer include transarterial chemoembolization, liver transplantation, partial hepatectomy, radio frequency ablation, and systemic treatment with Sorafenib [2, 3]. Currently, surgical resection is the primary HCC treatment for patients; metastasis and postoperative recurrence are major causes affecting patient prognosis [4]. Tumor metastasis and invasion usually led to early tumor recurrence. Thus, new targets to treat metastasis and recurrence of HCC are needed. The epithelial-mesenchymal transition (EMT) is important in HCC progression. Interfering with the EMT may reduce tumor invasion and metastasis, and improve patient prognosis. Nevertheless, the regulatory mechanisms remain unclear.

Circular RNAs (circRNAs) belong to a big family of endogenous non-coding RNAs (ncRNAs) that covalently link the 3’ and 5’ ends to form circular loops [5]. Previous investigations have suggested that circRNAs function in important biological processes of cancer cells, such as migration and invasion. Other studies have shown that circRNAs are indispensable molecules in gene expression regulation at the post-transcriptional level by functioning as microRNA (miRNA) sponges [6, 7]. For example, circRNA-5692 inhibits HCC progression by sponging miR-328-5p to promote DAB2IP expression [8]. hsa_circ_0000517 upregulation predicts an HCC adverse prognosis [9]. In our previous studies, we discovered that hsa_circ_0101145 is abnormally expressed in HCC, but its role has not yet been elucidated.

The present study showed that hsa_circ_0101145 is significantly upregulated in HCC cells and tissues. hsa_circ_0101145 also regulates the LAMC2-mediated EMT by functioning as an miR-548c-3p sponge. Thus, downregulation of hsa_circ_0101145 may have therapeutic potential in HCC treatment.

## RESULTS

### High hsa_circ_0101145 expression predicts poor HCC prognosis

**Figure 1:**
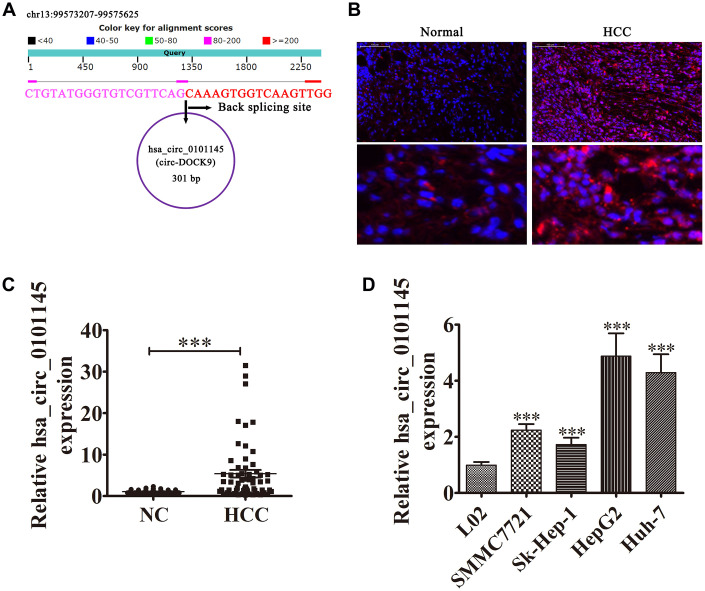
**Expression level and characteristics of the circular RNA hsa_circ_0101145.** (**A**) Genomic loci of the *HARS* gene and hsa_circ_0101145. (**B**) Expression and subcellular localization of hsa_circ_0101145 in HCC tissues and adjacent normal tissues was analyzed using *in situ* hybridization. (**C**) Expression of hsa_circ_0101145 in HCC tissues (60) and adjacent normal tissues (60) was detected using RT-qPCR. Data are presented as the mean ± SD. ^***^P < 0.001 vs. Normal. (**D**) hsa_circ_0101145 expression in HCC cells lines SMMC7721, Sk-Hep-1, HepG2, and Huh-7 and the human normal liver cell line L02 were analyzed using RT-qPCR. Data are presented as the mean ± SD. ^***^P < 0.001 vs. normal cells.

The hsa_circ_0101145 is derived from *DOCK9* gene exons, which are located at chr13:99573207-99575625. *DOCK9* consists of 24,186 bp and the spliced mature circRNA is 301 bp (Figure 1A). FISH revealed that hsa_circ_0101145 expression in HCC tissues was increased compared with adjacent normal tissues. The hsa_circ_0101145 was predominantly localized to the cytoplasm (Figure 1B). We then chose 60 pairs of human HCC and adjacent normal tissues for hsa_circ_0101145 expression analysis. qRT-PCR analysis confirmed that hsa_circ_0101145 expression increased in human HCC tissues compared with adjacent normal tissues (Figure 1C). We divided samples into high- (greater than adjacent normal tissues; n = 27) and low- (less than adjacent normal tissues; n = 33) expressing groups. There were no associations between hsa_circ_0101145 expression and clinical factors such as sex (female and male), patient age (≤ 50 years and > 50 years), and liver cirrhosis (negative and positive). Significant differences were found between TNM stage (I/II or III/IV, high), lymph node metastasis (positive and negative), and tumor size (≤ 5 cm and > 5 cm) in this study (Table 1), suggesting that hsa_circ_0101145 expression had a role in HCC progression. qRT-PCR revealed that hsa_circ_0101145 expression increased in the HCC cell lines Huh-7, Sk-Hep-1, SMMC7721, and HepG2 compared with the normal human liver cell line L02 (Figure 1D). HepG2 and Huh-7 cells had the highest hsa_circ_0101145 expression and were selected for further experiments.

**Table 1:** The correlation between hsa_circ_0101145 expression and clinicopathologic features of 60 HCC patients.

**Characteristics**	**Numbers**	**Expression of hsa_circ_0101145**		***P* value**
		**Low (N = 33)**	**High (N = 27)**	
**Sex**				0.644
male	32	17	15	
female	28	16	12	
**Age**				0.169
≤50	24	14	10	
>50	36	19	17	
**Liver cirrhosis**				0.321
Positive	42	20	22	
Negative	18	13	5	
**TNM stage**				0.016
I and II	29	22	7	
III and IV	31	11	20	
**Lymph node metastasis**				0.018
negative	40	27	13	
positive	20	6	14	
**Tumor size**				0.037
≤ 5 cm	43	26	17	
> 5 cm	17	7	10	

### Downregulation of hsa_circ_0101145 suppressed HCC proliferation *in vitro* and *in vivo*

**Figure 2:**
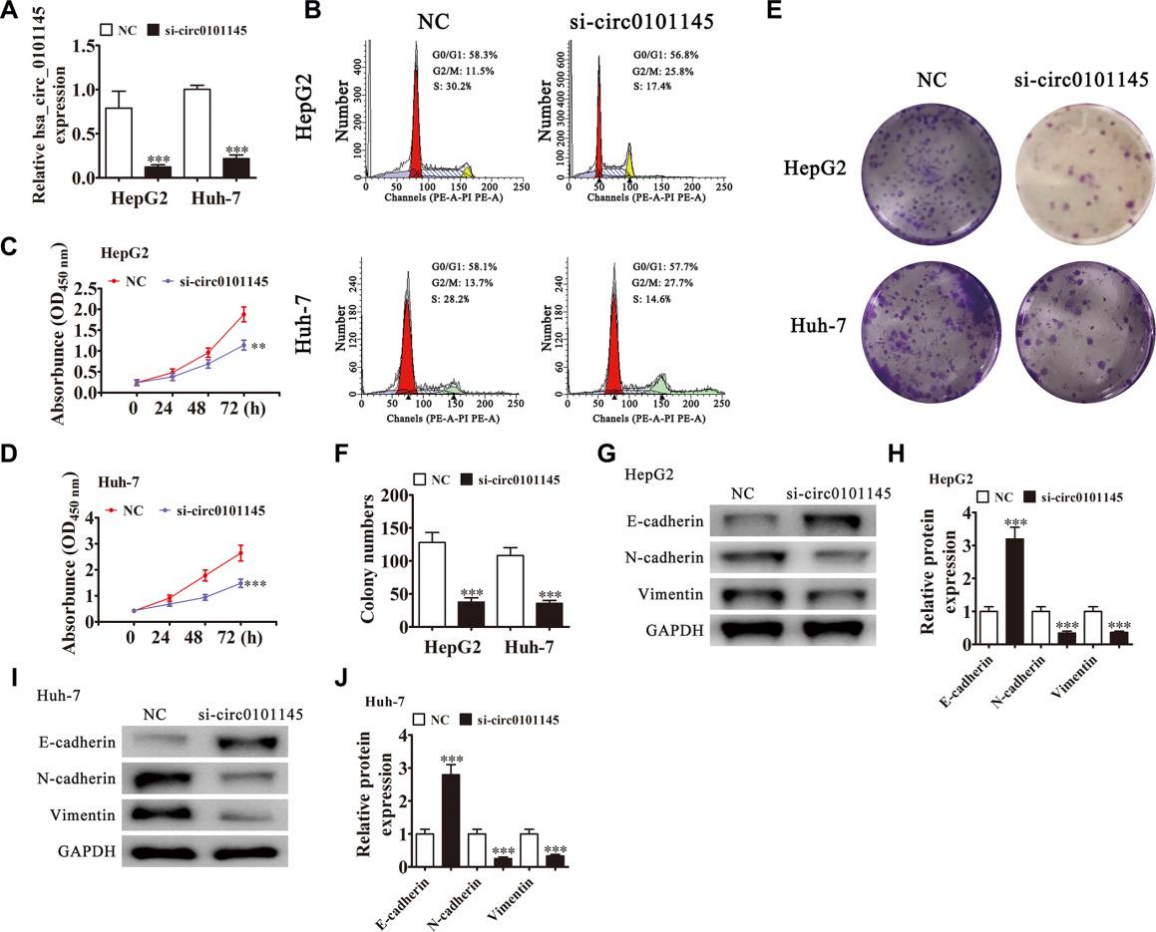
**Downregulation of hsa_circ_0101145 suppressed HCC cell proliferation *in vivo* and *in vitro*.** (**A**) RT-qPCR detection of hsa_circ_0101145 expression in HepG2 and Huh-7 cells following transfection with siRNA targeting hsa_circ_0101145 (si-circ0101145) or the negative control (NC). Data are presented as the mean ± SD. ^***^P < 0.001 vs. NC. (**B**) Flow cytometry showing the percentages of cells in G1, S, or G2 phase in HepG2 and Huh-7 cells. (**C** and **D**) CCK-8 assay showing the proliferation of HepG2 (**C**) and Huh-7 (**D**) cells. Data are presented as the mean ± SD. ^**^P < 0.01, ^***^P < 0.001 vs. NC. (**E** and **F**) Colony formation assay showing proliferation of HepG2 and Huh-7 cells. Data are presented as mean ± SD. ^***^P < 0.001 vs. NC. (**G**–**J**) Western blot analysis of the expression of E-cadherin (epithelial marker), and N-cadherin and vimentin (mesenchymal markers). Data are presented as the mean ± SD. ^***^P < 0.001 vs. NC.

RT-qPCR revealed that hsa_circ_0101145 expression was decreased in Huh-7 and HepG2 cells following transfection with siRNA against hsa_circ_0101145 (si-circ-0101145) compared with the negative control (NC) (Figure 2A). Cell cycle distribution analysis showed that the proportion of cells in S-phase was decreased significantly and that the G2/M-phase proportion was increased following depletion of hsa_circ_0101145 (Figure 2B), suggesting cell cycle arrest at G2/M. CCK-8 (Figure 2C and 2D) and colony formation (Figure 2E and 2F) assays demonstrated that hsa_circ_0101145 silencing decreased proliferation of both HepG2 and Huh-7 cells. Western blotting revealed that knockdown of hsa_circ_0101145 promoted epithelial marker E-cadherin expression, but decreased mesenchymal markers N-cadherin and vimentin expression (Figure 2G–2J), suggesting that depletion of hsa_circ_0101145 inhibited the EMT of HCC cells.

**Figure 3:**
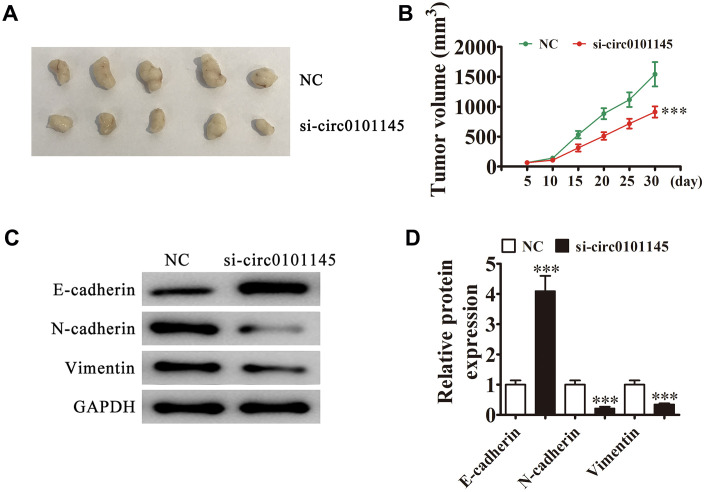
**Downregulation of hsa_circ_0101145 decreased HCC tumor formation in nude mouse xenografts.** (**A**) Representative images of nude mouse xenografts of HepG2 cells (n = 6). (**B**) Tumor volumes in mice were measured every 5 days. Data are means ± SD. ^***^P < 0.001 vs. NC. (**C** and **D**) Western blot analysis of the expression of E-cadherin (epithelial marker), and N-cadherin and vimentin (mesenchymal markers). Data are presented as the mean ± SD. ^***^P < 0.001 vs. NC.

We employed xenograft mouse models to verify the effects of hsa_circ_0101145 on tumor growth *in vivo*. We inoculated nude mice with hsa_circ_0101145-silenced (si-circ0101145) or NC HepG2 cells. After one month, we harvested the xenografts as demonstrated in Figure 3A. We detected smaller tumors in mice injected with si-circ0101145 HepG2 cells, but observed larger xenografts in mice inoculated with wild-type cells. Xenografts grew slower in volume in the si-circ0101145 group (Figure 3B). Western blotting revealed that knockdown of hsa_circ_0101145 promoted E-cadherin expression, but decreased N-cadherin and vimentin expression (Figure 3C and 3D). In summary, the above data showed that hsa_circ_0101145 knockdown suppressed HCC proliferation *in vitro* and *in vivo*.

### Downregulation of hsa_circ_0101145 suppressed tumor metastasis *in vitro* and *in vivo*

**Figure 4:**
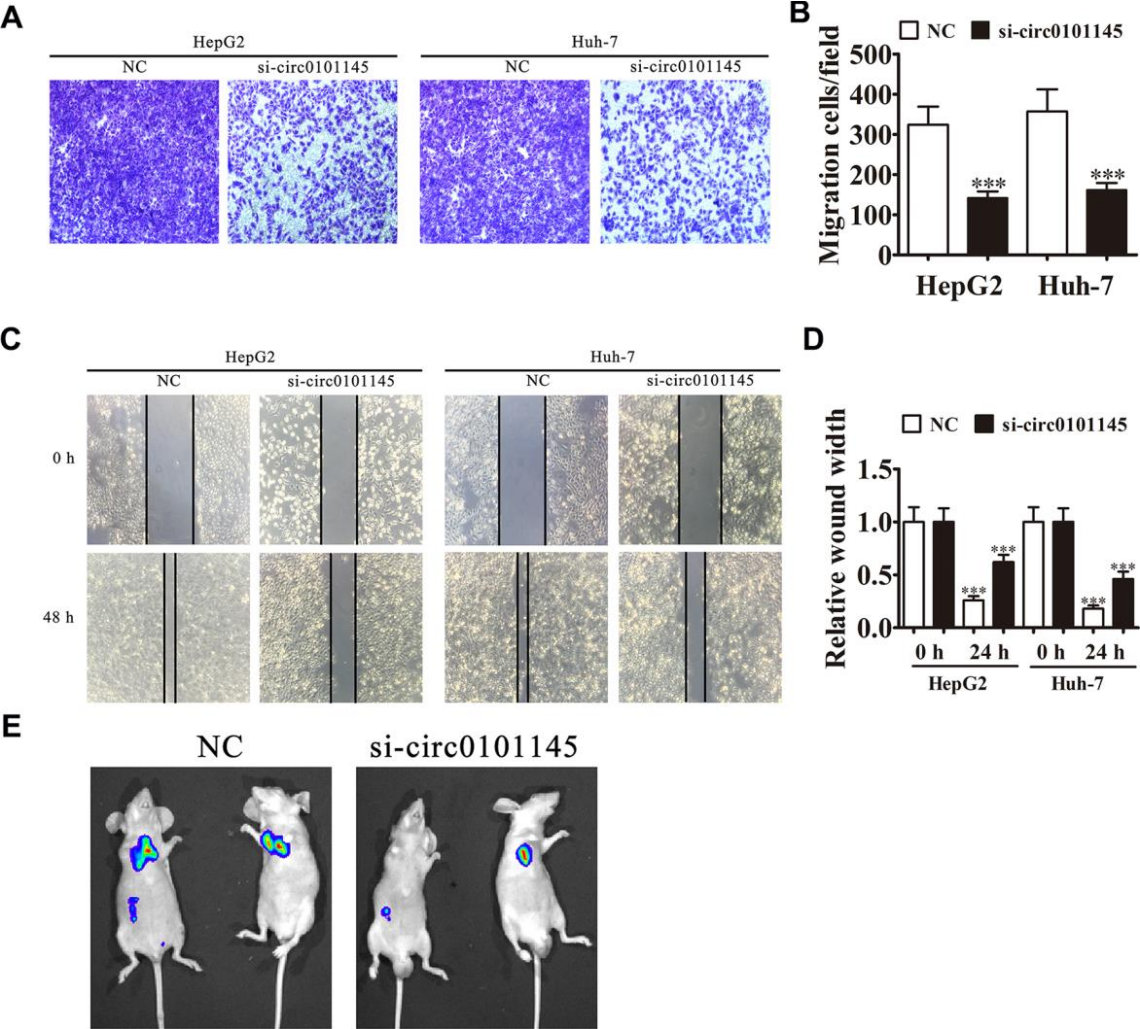
**Downregulation of hsa_circ_0101145 suppressed tumor metastasis *in vivo* and *in vitro*.** (**A** and **B**) Transwell cell migration assays in HepG2 and Huh-7 cells. Data are means ± SD. ^***^P < 0.001 vs. NC. (**C** and **D**) Wound-healing assays showing the effect of hsa_circ_0101145 on the closure of scratch wounds. Data are presented as the mean ± SD. ^***^P < 0.001 vs. NC. (**E**) Live imaging shows the effects of hsa_circ_0101145 on the metastasis of HepG2 cells 30 days after intravenous tail injection.

We then studied the effects of hsa_circ_0101145 on HCC invasiveness. *In vitro* experiments using the Transwell assay confirmed that hsa_circ_0101145 knockdown suppressed cell migration in both HepG2 and Huh-7 cells (Figure 4A and 4B). Wound-healing assays demonstrated that hsa_circ_0101145 depletion decreased the invasion of both HepG2 and Huh-7 cells (Figure 4C and 4D). Following 30 days of intravenous tail injection of HepG2 cells with or without hsa_circ_0101145 knockdown, the metastatic ability of HepG2 cells was decreased with hsa_circ_0101145 silencing, as determined by live cell imaging (Figure 4D). The data indicated that hsa_circ_0101145 knockdown suppressed HCC invasion both *in vitro* and *in vivo*.

### Interactions among hsa_circ_0101145, miR-548c-3p, and LAMC2

**Figure 5:**
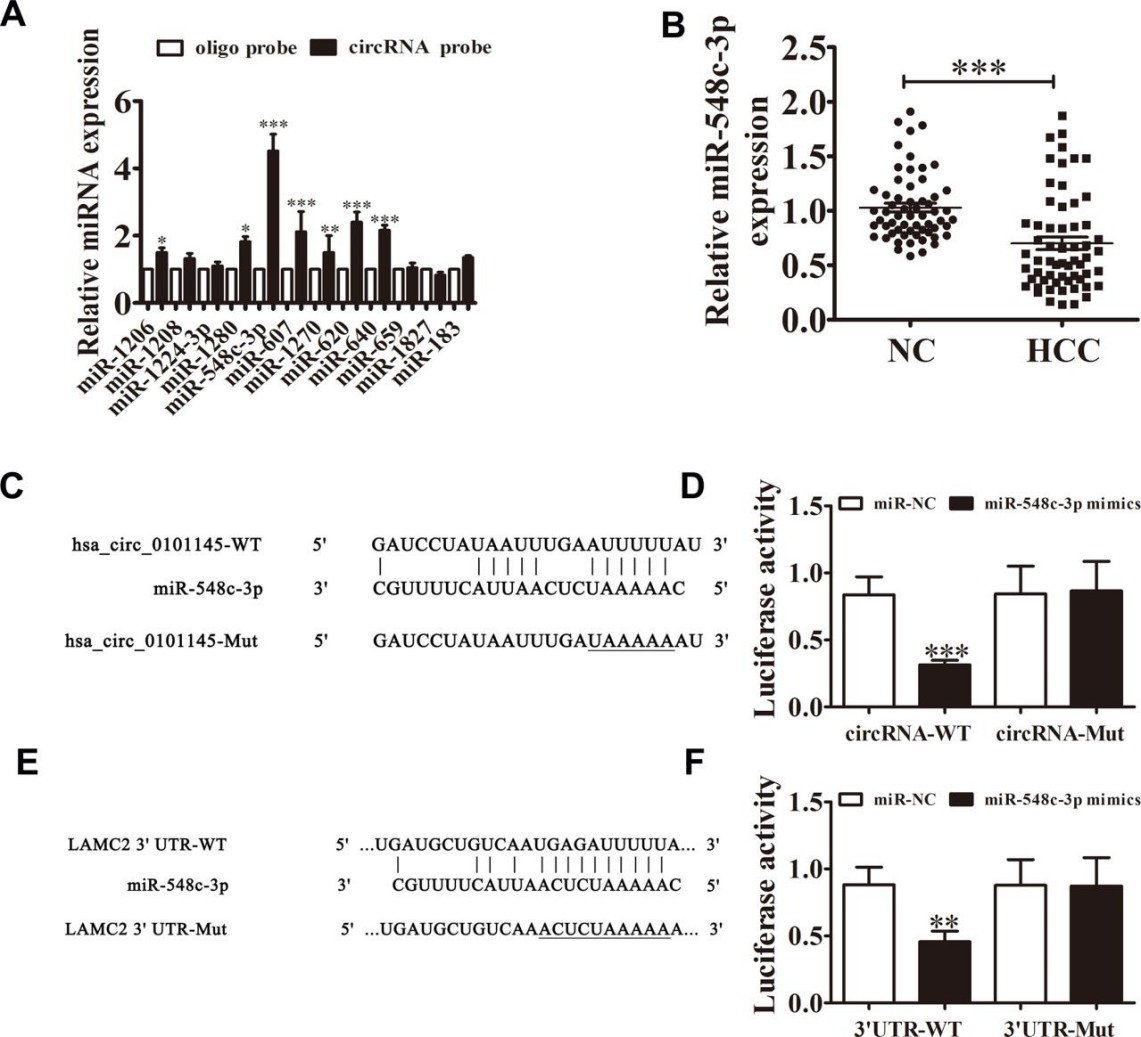
**Interactions among hsa_circ_0101145, miR-548c-3p, and LAMC2.** (**A**) RT-qPCR analyses revealed that miR-548c-3p was the only miRNA that was abundantly pulled down by the hsa_circ_0101145 probe in HepG2 cells. Data are presented as means ± SD. ^*^P < 0.05, ^**^P < 0.01, ^***^P < 0.001 vs oligo. (**B**) RT-qPCR shows the expression of miR-548c-3p in HCC tissues (60) and adjacent normal tissues (60). Data are presented as the mean ± SD. ^***^P < 0.001 vs. Normal. (**C**) Prediction of binding sites of miR-548c-3p in hsa_circ_0101145. The mutant version of hsa_circ_0101145 is presented. (**D**) Relative luciferase activity determined at 48 h after transfection of 293T cells with miR-548c-3p mimic/NC or hsa_circ_0101145 wild-type/Mut. Data are presented as means ± SD. ^***^P < 0.001. (**E**) Prediction of binding sites of miR-548c-3p in the 3'UTR of LAMC2. The mutant version of 3'UTR-LAMC2 is shown. (**F**) Relative luciferase activity determined at 48 h after transfection of 293T cells with miR-548c-3p mimic/NC or 3'UTR-LAMC2 wild-type/Mut. Data are presented as means ± SD. ^***^P < 0.001.

Bioinformatics analysis revealed that hsa_circ_0101145 can interact with different miRNAs such as miR-1260, miR-1280, miR-183, miR-607, and miR-548c-3p. RT-qPCR analyses showed that miR-548c-3p was the only miRNA that was abundantly expressed after RNA pulldown assay using the hsa_circ_0101145 probe in HepG2 cells (Figure 5A). RT-qPCR also showed that miR-548c-3p expression in HCC tissues decreased compared with adjacent normal tissues (Figure 5B).

Bioinformatics analysis validated that miR-548c-3p was a downstream target of hsa_circ_0101145. To validate the connection between miR-548c-3p and hsa_circ_0101145, we constructed mutated and wild-type hsa_circ_0101145 sequences including the miR-548c-3p binding sequence into a luciferase reporter vector (Figure 5C). We then transfected this luciferase reporter vector into 293T cells in the presence or absence of a miR-548c-3p mimic. Luciferase reporter assay results indicated that miR-548c-3p inhibited luciferase activity in wild-type cells but not in mutated cells (Figure 5D), indicated that miR-548c-3p is a target of hsa_circ_0101145. Bioinformatics analysis also revealed that LAMC2 is a downstream target of miR-548c-3p (Figure 5E); luciferase reporter analysis confirmed that miR-548c-3p can interact with the LAMC2 3’UTR (Figure 5F), suggesting that hsa_circ_0101145 promotes HCC cell invasion and proliferation by sponging miR-548c-3p.

### miR-548c-3p downregulation or LAMC2 overexpression restored the migration and proliferation ability of HepG2 and Huh-7 cells following silencing of hsa_circ_0101145

**Figure 6:**
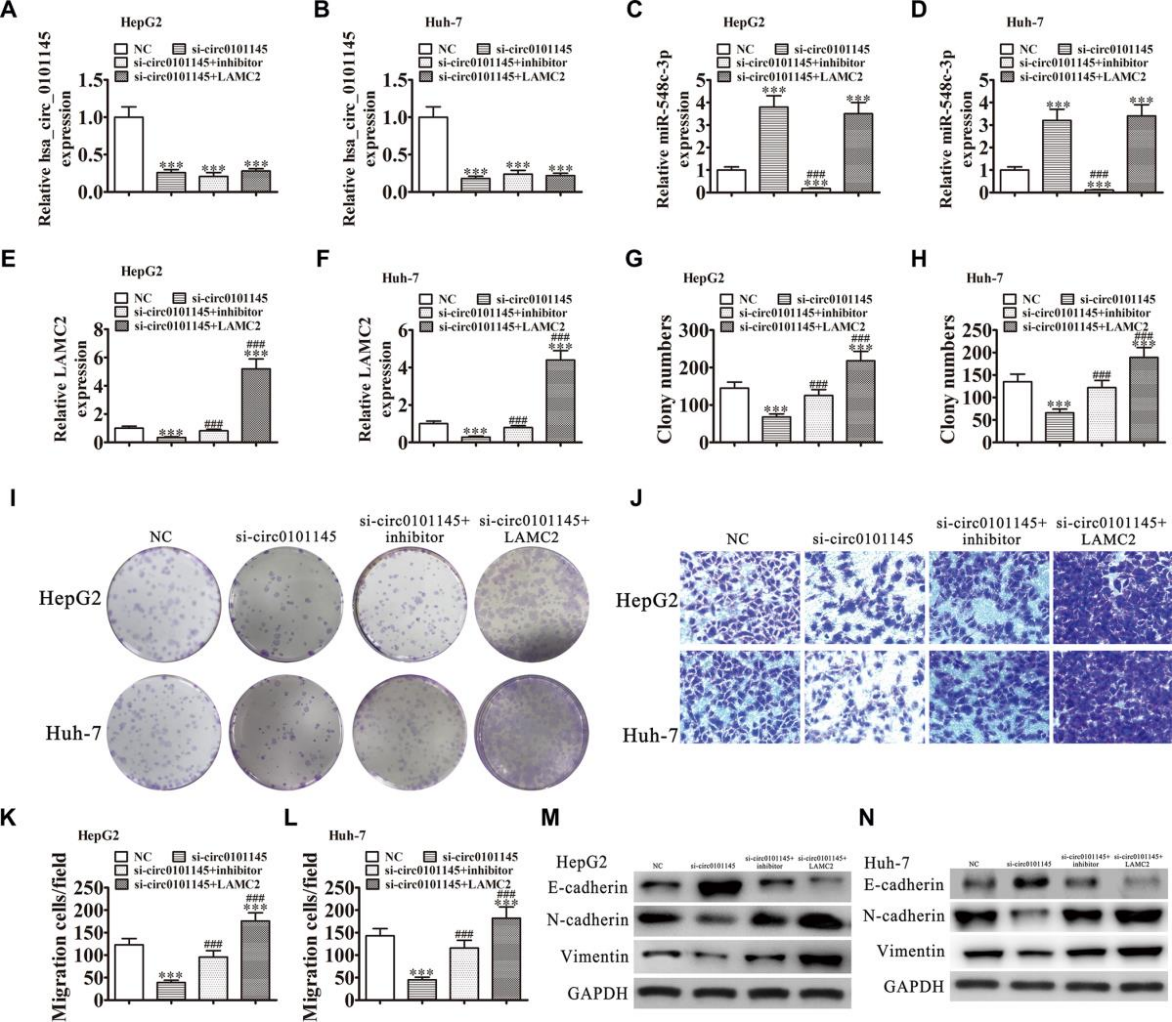
**Downregulation of miR-548c-3p or overexpression of LAMC2 restored the proliferation and migration abilities of both HepG2 and Huh-7 cells following silencing of hsa_circ_0101145.** (**A**–**F**) RT-qPCR analysis shows the expression of hsa_circ_0101145 (**A** and **B**), miR-548c-3p (**C** and **D**), and LAMC2 (**E** and **F**) in HepG2 and Huh-7 cells following transfection or treatment with NC, si-circ0101145, miR-548c-3p inhibitor, LAMC2 overexpression vector (LAMC2) single or combined. Data are presented as the mean ± SD. ^***^P < 0.001 vs. NC. ^###^P < 0.001 vs. si-circ0101145. (**G**–**I**) Cell proliferation was analyzed by colony formation. Data are presented as the mean ± SD. ^***^P < 0.001 vs. NC. ^###^P < 0.001 vs. si-circ0101145. (**J**–**L**) Cell migration was assessed in HepG2 and Huh-7 cells using Transwell assays. Data are presented as the mean ± SD. ^***^P < 0.001 vs. NC. ^###^P < 0.001 vs. si-circ0101145. (**M** and **N**) Western blot analysis of the expression of E-cadherin, N-cadherin, and vimentin.

Downregulation of hsa_circ_0101145 was confirmed by RT-qPCR. Downregulation of miR-548c-3p or LAMC2 overexpression did not restore hsa_circ_0101145 levels in Huh-7 or HepG2 cells (Figure 6A and 6B), suggesting that both miR-548c-3p and LAMC2 are downstream targets of hsa_circ_0101145. We also found that hsa_circ_0101145 silencing promoted miR-548c-3p expression, while treatment with a miR-548c-3p specific inhibitor or miR-548c-3p expression even after silencing of hsa_circ_0101145. Overexpression of LAMC2 did not influence miR-548c-3p expression (Figure 6C and 6D). RT-qPCR showed that silencing of hsa_circ_0101145 decreased LAMC2 expression while inhibition of miR-548c-3p restored the levels of LAMC2 following silencing of hsa_circ_0101145 in both HepG2 and Huh-7 cells (Figure 6E and 6F). These results confirmed that hsa_circ_0101145 regulated LAMC2 expression by sponging miR-548c-3p. Colony formation (Figure 6G–6I) and Transwell (Figure 6I–6L) assays showed that miR-548c-3p downregulation or LAMC2 overexpression recovered the migration and proliferation abilities of both Huh-7 and HepG2 cells after hsa_circ_0101145 silencing, suggesting that hsa_circ_0101145 promoted HCLC cell migration and proliferation by sponging miR-548c-3p. Western blot analysis demonstrated that miR-548c-3p downregulation or LAMC2 overexpression recovered the EMT in both HepG2 (Figure 6M) and Huh-7 (Figure 6N) cells after hsa_circ_0101145 silencing.

### LAMC2 overexpression reversed miR-548c-3p-induced cell growth and migration inhibition *in vitro*

**Figure 7:**
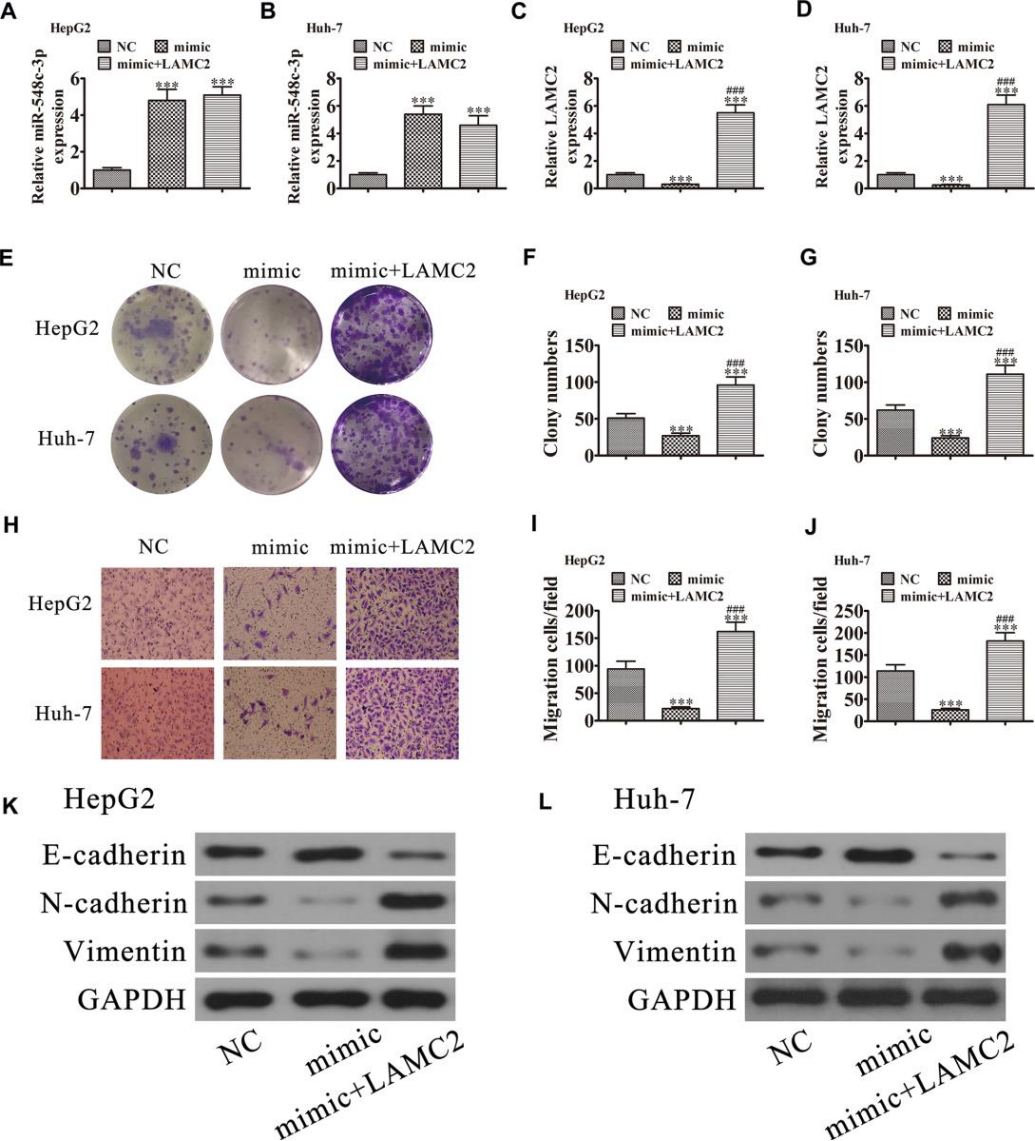
**LAMC2 overexpression reversed miR-548c-3p-induced cell migration and growth inhibition *in vitro*.** HepG2 and Huh-7 cells were transfected with miR-548c-3p mimics with or without the LAMC2 overexpression vector. (**A** and **B**) RT-qPCR assay showing the expression of miR-548c-3p (**A** and **B**) and LAMC2 (**C** and **D**) in HepG2 and Huh-7 cells. Data are mean ± SD. ^***^P < 0.001 versus NC. ^###^P < 0.001 versus mimic. (**E**–**G**) Colony formation assay showing the proliferation of HepG2 and Huh-7 cells. Data are mean ± SD. ^***^P < 0.001. ^###^P < 0.001 versus mimic. (**H**–**J**) Cell migration were assessed in HepG2 and Huh-7 cells by Transwell assays. Data are mean ± SD. ^***^P < 0.001 versus NC. ^###^P < 0.001 versus mimic. (**K** and **L**) Western blot analysis of the expression of E-cadherin, N-cadherin, and vimentin. Data are mean ± SD. ***P < 0.001 vs. NC. ^###^P < 0.001 vs. the mimic.

RT-qPCR demonstrated that miR-548c-3p expression was increased in Huh-7 and HepG2 cells following transfection with miR-548c-3p mimics. Upregulation of LAMC2 could not restore miR-548c-3p expression (Figure 7A and 7B), suggesting that LAMC2 is a downstream target of miR-548c-3p. RT-qPCR revealed that miR-548c-3p overexpression decreased LAMC2 expression in HepG2 and Huh-7 cells, but after transfection with a LAMC2 overexpression vector promoted LAMC2 expression (Figure 7B and 7C). Colony formation (Figure 7E–7G) and Transwell (Figure 7H–7J) assays illustrated that LAMC2 overexpression restored HepG2 and Huh-7 cell proliferation and migration abilities following upregulation of miR-548c-3p. Western blot analysis showed that upregulation of LAMC2 recovered the EMT in both HepG2 (Figure 7K) and Huh-7 (Figure 7L) cells after overexpression of miR-548c-3p.

## DISCUSSION

Growing evidence has indicated that the circRNA/miRNA/mRNA regulatory system plays a novel and important regulatory role in the progression of many cancers, such as gastric cancer [11], breast cancer [12], pancreatic carcinoma [13], cervical cancer [14], ovarian cancer [15], and HCC [16]. They are validated to facilitate proliferation, metastasis, and chemotherapeutic resistance [17, 18]. Previous studies have found that circMTO1 functions as an miR-9 sponge to suppress HCC progression [10]. We also discovered that hsa_circ_0101145 was abnormally expressed in HCC, but the role of hsa_circ_0101145 in HCC is unknown.

The present study suggested that hsa_circ_0101145 expression increased significantly in both HCC cell lines and tissues and the increased hsa_circ_0101145 expression resulted in poor prognosis. The data also indicated that hsa_circ_0101145 downregulation decreased HCC cell migration and proliferation, and the EMT. Decreased expression of hsa_circ_0101145 abated the EMT via increased E-cadherin expression and decreased N-cadherin as well as vimentin expression. The competing endogenous RNA (ceRNA) hypothesis states that circRNAs, lncRNAs, mRNAs, and pseudogenes communicate with and regulate one another’s expression by combining with shared miRNAs response elements, which provides a novel mechanism of post-transcriptional regulatory networks [19]. We also found that miR-548c-3p was a downstream target of hsa_circ_0101145. Previous studies have indicated that miR-548c-3p expression can inhibit the progression of many cancers, including papillary thyroid carcinoma [20], breast cancer [21], and osteosarcoma [22]. We found that miR-548c-3p expression decreased in HCC tissues. hsa_circ_0101145 downregulation promoted miR-548c-3p expression, while miR-548c-3p downregulation restored HCC cell proliferation, and migration, and the EMT following silencing of hsa_circ_0101145. Luciferase reporter and RNA pulldown assays also confirmed that hsa_circ_0101145 can interact with miR-548c-3p.

circRNAs regulate miRNA target gene expression by functioning as a ceRNA. We discovered that LAMC2 was a putative miR-548c-3p target via TargetScan. The luciferase reporter assay confirmed that miR-548c-3p interacted with LAMC2 3’UTR, which is also known as the laminin γ2 chain gene. Recent results suggest that LAMC2 could function as an oncogene to enhance the progression of many types of cancer, including penile squamous cell carcinoma [23], esophageal squamous cell carcinoma [24], gastric cancer [25], tongue squamous cell carcinoma [26], and colorectal cancer [27]. Silencing of LAMC2 reversed the EMT, invasion, and metastasis abilities [28, 29]. The present study also showed that overexpression of LAMC2 restored HCC cell migration and proliferation, and the EMT following hsa_circ_0101145 silencing or miR-548c-3p overexpression, suggesting that hsa_circ_0101145 silencing reversed the EMT in HCC through miR-548c-3p/LAMC2 axis regulation.

## CONCLUSION

Our findings indicate that hsa_circ_0101145 regulates LAMC2 expression by functioning as a miR-548c-3p decoy, leading to HCC development and tumorigenesis. Our data also suggest that hsa_circ_0101145 is a potentially novel prognostic, diagnostic, and therapeutic target for HCC. The hsa_circ_0101145/miR-548c-3p/LAMC2 regulatory network supplies an enhanced knowledge of the mechanism underlying HCC progression and pathogenesis.

## MATERIALS AND METHODS

### Ethics statement

We employed 12 four-week-old BALB/c nude mice weighing 15~20 g (SLARC, Shanghai, China) in the present study. The ethics committee at The First Hospital of Jilin University approved the animal experiments.

### Tissue samples

We collected 60 fresh HCC tissues as well as adjacent noncancerous tissues from patients enrolled in Xinhua Hospital affiliated with Shanghai Jiaotong University. The tissue samples were immediately frozen in liquid nitrogen and stored at -80°C. Clinicopathological features of the HCC patients are shown in Table 1. We obtained written informed consent from each patient. Sir Run Run Shaw Hospital, affiliated with Zhejiang University, approved the experimental protocols in this study.

### Cell culture and cell lines

We purchased human HCC cell lines (HepG2, SMMC7721, Huh-7, and Sk-Hep-1) and a normal human liver cell line (L02) from the American Tissue Culture Collection (Manassas, VA, USA). We cultured cells in Roswell Park Memorial Institute 1640 medium (HyClone, Logan, UT, USA) containing 10% fetal bovine serum (FBS; Gibco, Gaithersburg, MD, USA) in a humidified atmosphere with 5% CO_2_ at 37°C. We transfected miR-548c-3p inhibitors, lentiviral-stabilized hsa_circ_0101145 silenced vector (si-circ0101145), miR-548c-3p mimics, LAMC2 overexpression vector (FOSL2), and the negative controls (NCs) into cultured Huh-7 and HepG2 cells prior to subsequent experiments. We purchased lentiviral-based short hairpin RNA (shRNA) targeting hsa_circ_0101145 and lentiviruses overexpressing LAMC2 from GeneChem (Shanghai, China).

### Bioinformatic analyses

We predicted circRNA/miRNA target genes using the tool available on the Circular RNA Interactome website. We predicted interactions between miRNAs and mRNAs via TargetScan (http://www.targetscan.org/).

### Flow cytometry analysis of the cell cycle

We fixed cells in 70% ethanol overnight at 4°C. We resuspended fixed cells in staining solution (Beyotime, Shanghai, China) and incubated them for 30 min at 4°C. Stained cells were analyzed using flow cytometry (Beckman Coulter, Brea, CA, USA).

### Fluorescence *in situ* hybridization (FISH)

We used hsa_circ_0101145-specific FITC-labeled probes for *in situ* hybridization. We counterstained nuclei with 4,6-diamidino-2-phenylindole (DAPI) and performed FISH according to the standard protocol (Genepharma, Shanghai, China). The hsa_circ_0101145 probe sequence was as follows: 5’- GGTGTCGTTCAGCAAAGTGGTCAAG -3’.

### Cell proliferation assay

We detected Huh-7 and HepG2 cell proliferation using the Cell Counting Kit-8 (CCK-8) assay according to standard protocol (Invitrogen, Carlsbad, CA, USA). Briefly, we seeded 2000 cells in 100 μL in different groups into 96 wells. Following the addition of 10 μl of CCK-8, we detected cell viability at 0, 24, 48, and 72 h.

We seeded Huh-7 and HepG2 cells from different groups at a density of 2000 cells per well into plates with six wells for colony formation assays and cultured cells with Dulbecco’s modified Eagle medium (DMEM) containing 10% FBS for 10 days. Cells were washed with phosphate-buffered saline, fixed with 4% paraformaldehyde for 30 min, stained with crystal violet, and counted.

### qRT-PCR

We extracted RNA using TRIzol reagent (Invitrogen) and synthesized cDNA with a pTRUEscript First Strand cDNA Synthesis Kit (Aidlab, Beijing, China). We performed qRT-PCR with 2× SYBR Green qPCR Mix (Invitrogen) with the ABI 7900HT qPCR system (Thermo Fisher Scientific, Waltham, MA, USA). We determined fold-changes in expression via the 2^−ΔΔCT^ method. We performed qRT-PCR amplification with the following primers: hsa_circ_0101145: forward, 5’- GAGCTGGA TTATCAAGG -3’, reverse, 5’- GATCAATGGCGG AATAAGCAG -3’; miR-548c-3p: forward, 5’- ACACTC CAGCTGGGCAAAAATCTCAAT -3’, reverse, 5’- CTC AACTGGTGTCGTGGA -3’; LAMC2: forward, 5’- TAC CAGAGCCAAGAACGCTG -3’, reverse, 5’- CGCAGTT GGCTGTTGATCTG -3’; U6: forward, 5’- CTCGCTTCG GCAGCACA -3’, reverse, 5’- AACGCTTCATTTGCGT -3’; and glyceraldehyde 3-phosphate dehydrogenase (GAPDH): forward, 5’- AATCCCATCACCATCTTCC- 3’, reverse: 5’- CATCACGCCACAGTTTCC -3’. We normalized LAMC2 and hsa_circ_0101145 expression levels to GAPDH; and miR-548c-3p expression to U6.

### Wound healing assay

We seeded Huh-7 and HepG2 cells transfected with the relevant vectors into six-well plates to form a single confluent cell layer. Wounds were generated with a 100 μL tip. At 0 and 2 days after wound scratching, we photographed wound widths using a phase contrast microscope.

### Transwell assays

We suspended 2 × 10^4^ cells in 200 μL serum-free culture medium and placed them in upper chamber to perform a Transwell migration assay. We precoated the Transwell chamber with Matrigel (BD Biosciences, San Jose, CA, USA), and added an equivalent amount of cells to upper chamber for the invasion assay. Then, we added 500 μL of DMEM containing 15% FBS to lower chamber. We removed the cells in upper chamber after incubation for 1 day, and fixed the cells that invaded or migrated to the lower membrane surface with 4% paraformaldehyde followed by staining with 0.1% Crystal Violet solution. We counted and photographed invaded or migrated cells.

### RNA pulldown assay

We performed an RNA pulldown assay as described previously with minor modifications [10]. A biotin-labeled hsa_circ_0101145 probe was synthesized by Genepharma; the probe sequence was complementary to the back-spliced junction of hsa_circ_0101145. We lysed, scratched, and sonicated hsa_circ_0101145-overexpressing HepG2 cells. After centrifugation, we retained 50 μL supernatant as the input, and incubated the remaining with streptavidin Dynabeads (M-280, Invitrogen) conjugated with biotin-labeled hsa_circ_0101145 probe overnight at 4°C. After washing, we isolated total RNA and determined miRNA expression by RT-qPCR.

### Dual-luciferase reporter assay

We co-transfected 293T cells with 150 ng empty pmiR-GLO-NC or pmiR-GLO-circ-0101145-wt or with pmiR-GLO-circ-0101145-mut, pmiR-GLO-LAMC2-wt, or pmiR-GLO-LAMC2-mut (Sangon Biotech, China) as well as with 2 ng internal control pRL-TK (Promega, Madison, WI, USA). We also cotransfected 293T cells with pPG-miR-NC or pPG-miR-548c-3p. We used the dual-luciferase reporter assay kit to assess luciferase activity according to standard protocol. We normalized relative luciferase activity to Renilla luciferase activity.

### Western blots

We extracted protein from tumor tissues or cells via RIPA lysis buffer (Sigma-Aldrich, St. Louis, MO, USA). Protein concentrations were standardized with the BCA Protein Assay kit prior to SDS-PAGE and transfer to nitrocellulose membranes. After blocking with 5% non-fat milk and incubation with primary antibodies, we incubated membranes with the corresponding horseradish peroxidase-conjugated secondary antibody. GAPDH was employed as an internal control.

### Metastasis assays and tumor xenograft formation

We injected 2 × 10^7^ HepG2 cells with or without hsa_circ_0101145 silencing into a nude mouse right flank. We measured tumors (volume = 1/2 × length × width^2^) using a vernier caliper every 5 days for 1 month before euthanizing the mice.

For metastasis analysis, we intravenously injected 2 × 10^5^ HepG2 cells transfected with the luciferase expression vector with or without hsa_circ_0101145 silencing into mice tails. We analyzed HepG2 cell metastasis by bioluminescence imaging following intravenous injection (150 mg luciferin/kg body weight) of luciferin for 1 month.

### Statistical analysis

We analyzed data using GraphPad Prism (GraphPad Software Inc., La Jolla, CA, USA). We performed overall survival analysis by Kaplan–Meier curves and log-rank test for significance. We used two-tailed Student’s *t*-tests to determine statistical significance in two groups and employed one-way ANOVA with post hoc Bonferroni test for three or more groups. We analyzed correlations via Pearson correlation test. We reported data as means ± standard deviation (SD). P < 0.05 indicated statistical significance.
